# A value approach to measuring the effect on health resource usage of specialist cardiac interventions in Wales

**DOI:** 10.23889/ijpds.v9i5.1661

**Published:** 2024-09-18

**Authors:** Gareth Davies, Ashley Akbari, Rowena Bailey, Lloyd Evans, Kendal Smith, Richard Palmer, Jonathan Goodfellow, Michael Thomas, Kerryn Lutchman-Singh

**Affiliations:** 1 Swansea University; 2 NHS Wales; 3 Welsh Health Specialised Services Committee; 4 Hywel Dda University Health Board

## Objective

The Welsh Health Specialised Services Committee wanted to demonstrate how specialised interventions reduced subsequent pressures on the health services.

We analysed the variation in the cost of patient pathways for eight cardiac interventions in both planned and emergency settings. A standard cost was allocated to each health event (hospital admissions, general practitioner interactions, outpatient attendances and accident and emergency attendances). A comparison was made for each intervention relating to the condition before the intervention against costs afterwards. In addition, analysis was carried out measuring which patient's characteristics affected the likelihood that patients incurred higher post-intervention costs.

## Results

10,480 interventions were analysed. In 9 (of the 16) intervention combinations, post-intervention costs dropped. After the implantation of a Cardiac Device, health resource usage dropped by £1,461 per annum. In addition, the likelihood of falling into a higher cost category increased depending on deprivation. For percutaneous coronary intervention (PCI) the odds ratio and 95% confidence interval was 1.34 (1.03 to 1.76) and for Cardiac Devices 2.91 (1 to 15.23).

## Conclusion

Some interventions in Cardiac realise significant post intervention savings for the health service. Deprivation affects the risk of patients falling into high post-intervention cost categories for PCI and Cardiac Devices.

By using anonymised population-scale linked individual-level data, it is possible to highlight interventions that as well as benefiting patients successfully, reduce future health resource usage. This type of analysis can also highlight how deprivation is linked to increased post intervention costs.

